# Mr. Crowfoot, on Apoplexy, in Explanation

**Published:** 1802-08-01

**Authors:** W. Crowfoot

**Affiliations:** Beccles


### *To the Editors of the Medical and Phyfical Journal.*

Gentlemen,

THE *two words* which Dr. Langflow denies to have made ufe of, were, *considerable* and *blood*; and *not*, as I have by miftake mentioned, *extravasation*. It is my duty, under this conviction, to repair the wrong, if any has been committed; and to requeft that you will explain it to your Readers in your next Publication.

I am not fenfible, however, that I have done any injuftice to Dr. L. for the term *extravasation* is virtually *denied* by a definition perfectly at variance with its common acceptation, and introduced for very obvious reasons;* and the two words above quoted, will appear to have been employed, by what I have already advanced, in the last Number of the Medical Journal. But should any question remain in your minds, I will beg leave to subjoin the following additional illustration; and if you think your Readers may, for want of this evidence, entertain the smallest doubt of my veracity, I hope, in vindication of my character, you will have the goodness to publish it: For I have nothing more at heart than the confirmation of *truth*, which is, and ought to be, the object of all rational disputation.

If, as Dr. L. declares, " he never did consider the extravasation as *considerable in this instance*, nor ever thought the compressing fluid *blood*," it is incumbent upon him to reconcile what follows with that idea. " Mr. C. proceeds to state (says Dr. L.) that in his judgment, if *considerable* extravasation had taken place, the most formidable symptoms would have arisen." Now, adds the Doctor, " I ask what more formidable could arise?" Surely, the remark upon this passage is very strong evidence that Dr. L. thought the extravasation *considerable in this instance*.

Dr. L. says, " I believed Mr. P. right in his opinion, that the attack was apoplectic, and that there was or had been extravasation, which had been the cause of it; that I highly approved of the bleeding and application of the blisters; for blisters (says Dr. L. in a note) have a tendency to take off or diminish *hæmorrhagic* diathesis."

This I conclude will be thought satisfactory proof, that the Doctor *did*, *in this instance*, consider the compressing fluid *blood*.

And the opinion that real Apoplexy was *always* occasioned by effusion of blood or serum, and generally of *blood*, is expressed repeatedly in his answer, as may be seen No. XXXIV. p. 553 to 563.

If I do not trespass too much, I would willingly obviate some misconstructions which appear to have been adopted by your Correspondents, from too implicit a reliance upon the quotations of my opponent; though the paper from whence the extracts

---

* The Reader is particularly referred to the term Extravasation, as given by Dr. L. in his answer, No. XXXIV. p. 561; and he is requested to compare it with the same word, as *explained* in No. XXXV. Medical Journal, p. 77. And for the *inconstant use of words*, vide Locke's Essay on Human Understanding, 17th Edition, 8vo. Vol. II. p. 91 and 92.

tracts are taken immediately precedes the anfwer, in the fame Journal.

The firft is thus expreffed as my pofition. "Mr. C. proceeds to ftate, that in his judgment, if confiderable extravafation had taken place, the moft formidable fymptoms *must have arifen*, &c. &c." Hence the Reader fuppofes this reafoning took place during the fit; whereas, it is neceffary to recollect, it was in a fubfequent ftage, or when the patient was fenfible, collected, and had no other complaint befides head-ach and ficknefs. My words will be found to be thefe: "I did not agree with him, by any means, in his doctrine of Extravafation; for in my judgment, a *considerable* extravafation, and confequently confiderable compreffion, muft produce fymptoms of the moft formidable nature, and which fymptoms, in the cafe above-ftairs, *did not appear to exist*."

It is therefore incontrovertible, that I was fpeaking of the recovered fituation of the patient (for indeed the paroxyfm was over before either Dr. L. or myfelf arrived) and of the ftate in which we found the lady; when, had there been *considerable* extravafation of blood upon the brain, we fhould have witneffed the ufual figns of *compression*, which *in this case* did not appear to exift.

One more inftance fhall fuffice. "I do remember (fays Dr. L.) ufing the two words Extravafation and Exudation; but Mr. C. fays, thefe two words convey a diftinction without a difference."

In the Statement, you will find, "I told the Doctor, he might call it which he pleafed; if it was *considerable*, compreffion muft be the confequence, and in my opinion it was a diftinction without a difference." My meaning, therefore, without torturing the expreffion, was plainly this—*Compression*, whether arifing from *considerable* extravafation of blood or ferum, was ftill compreffion, and muft have fhewn itfelf by its concomitant fymptoms.

I am forry to have taken up a fingle page of your valuable Journal with a matter merely perfonal; but I think you will allow the propriety, if not the neceffity, to repel a charge fo difgraceful, as the deliberate violation of *Truth*; and as the circulation of your Journal is not confined to this kingdom, or even to Europe, it is moft defirable to place my defence in that quarter where the calumny may be found.

I am, &c.

*Beccles, April* 8, 1802. W. CROWFOOT.

